# Artery of Percheron in the Differential Diagnosis of Acute Altered Mental Status

**DOI:** 10.1155/2021/5583248

**Published:** 2021-10-22

**Authors:** Maria Isabel Pérez Pan˜art, Beatriz Sierra Bergua

**Affiliations:** Emergency Department of Hospital, Clinico Universitario Lozano Blesa, Zaragoza, Spain

## Abstract

Bilateral thalamic infarction is a rare entity (it occurs in 0.6% of ischemic strokes) and can be confused with other vascular etiologies such as basilar syndrome and deep cerebral venous thrombosis, as well as neoplasms, infections, or toxins. It is typically characterized by the triad of altered mental status, vertical gaze paralysis, and memory impairment, although their symptoms can be highly variable. We describe the case of a young man who came to the emergency department presenting diplopia, speech alteration, and decreased level of consciousness with frequent fluctuations. Baseline computerized tomography was normal, and because of the clinical findings, thrombolysis was performed. Subsequently made magnetic resonance showed a bilateral acute thalamic infarction caused by an obstruction of the Percheron artery. Although Percheron syndrome has been previously described in medical journals, our case is an unusual case in which we could perform an acute intravenous thrombolytic treatment. Besides, it is important for emergency physicians to enclose the Percheron syndrome in the differential diagnosis of coma in young people so that emergent treatments such as thrombolysis can be employed.

## 1. Introduction

Thalamic vascularization is complex and varies from person to person. The paramedian or Percheron artery is a rare variant where a single thalamoperforant artery supplies both thalamic nuclei, with variable midbrain irrigation. Bilateral thalamic infarcts are rare, occurring in 0.6% of ischemic strokes and between 4 and 18% of all thalamic infarcts. [1].

This type of stroke is related to embolic phenomena, and cardioembolism should always be considered as part of its etiology, as well as the discovery of some congenital defects not known previously objectified. [2].

It is characterized by decreased consciousness, neuropsychological and memory impairment, and vertical gaze palsy. Oculomotor disturbances, hemiplegia, cerebellar ataxia, and movement disorders have also been described. It can mimic other pathological processes including intracranial haemorrhage, infection, and inflammation. [3].

Emergency physicians should be aware of this syndrome so that emergent treatments such as thrombolysis can be employed. However, diagnosis of Percheron artery infarction is often made in the late stage.

## 2. Case Report

A 43-year-old man with migraine as medical history for which he did not take any active pharmacological treatment begins two hours before his arrival in the emergency room with diplopia, speech alteration, and decreased level of consciousness with frequent fluctuations. He denied the consumption of any associated toxics.

The physical examination revealed predominantly femoral right hemiparesis, binocular strabismus with limited convergence and vertical eye movements, nonreactive miotic pupils, and fluctuations in the level of consciousness with a score on the Glasgow Scale that varied between 8 and 10.

**Figure 1:**
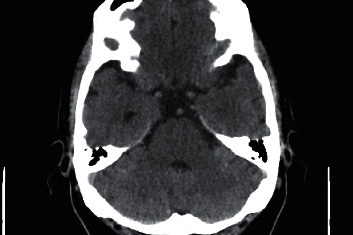
Baseline computerized axial tomography image without acute changes.

Among the complementary tests that were requested, the analyses showed no alterations (including toxic and drug profiles) and the baseline computerized axial tomography (TC) showed no haemorrhage or incipient signs of ischemia (Figure 1).

**Figure 2:**
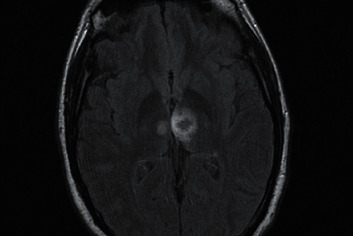
Magnetic resonance imaging where a bilateral acute thalamic infarction compatible with Percheron syndrome is observed appreciating hemorrhagic transformation in the left thalamus.

**Figure 3:**
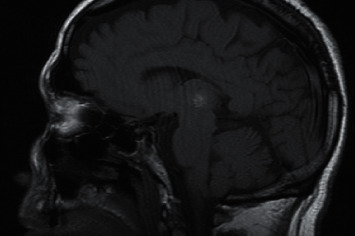
Magnetic resonance imaging where we can observe hemorrhagic transformation in the left thalamus of the infarction.

During his stay, the fluctuation in the level of consciousness persists and a right hemiparesis de novo is observed. Suspecting a posterior circulation stroke and in the absence of contraindications, intravenous thrombolysis was performed. The study was completed with the performance of computerized angiography that revealed a bilateral thalamic hypodensity predominantly in the posterior left thalamus (bitalamic infarction, probably Percheron) which was confirmed with the performance of magnetic resonance imaging (Figures 2 and 3), where a bilateral acute thalamic infarction compatible with Percheron syndrome is finally observed, appreciating hemorrhagic transformation in the left thalamus.

Due to the persistence of the alteration in the level of consciousness, the patient was admitted to the intensive care unit (ICU) for neurological control. During his stay in the ICU, the patient fully recovered his level of consciousness, persisting only at discharge a binocular diplopia of less intensity.

The study was completed with a study of autoimmunity, vitamin B12 and folic acid, serologies for brucellosis, human immunodeficiency virus, and syphilis that did not show any alterations and the performance of a regulated echocardiogram which revealed a patent foramen ovale, without intracardiac thrombosis.

## 3. Discussion

The artery of Percheron (AOP) is a rare variant of the paramedian arterial supply in which a single dominant thalamoperforating artery nourishes both the paramedian thalamus and, in some cases, the rostral midbrain. [4].

Bilateral thalamic infarcts are rare, occurring in 0.6% of ischemic strokes and between 4 and 18% of all thalamic infarcts. They are typically characterized by the triad of altered mental status, vertical gaze paralysis, and memory impairment, although their symptoms can be highly variable, including aphasia, amnesia, and hypersomnolence [1], as happened in our patient, where the fluctuation in the level of consciousness and diplopia were present from the beginning. Furthermore, our patient presented right hemiparesis. As we have seen in some publications such as Arauz et al. [5], motor paresis has been described as a frequent symptom and it may be explained by the cortical spinal tract impairment at the midbrain level.

Occlusion of the AOP is not the only condition that can be found in thalamic lesions. We want to highlight the importance of framing a syndrome within the differential diagnosis of altered level of consciousness in young people, among which we find other vascular etiologies such as basilar syndrome and deep cerebral venous thrombosis; pathologies such as Wernicke's encephalopathy, neoplasms, infections, Wilson's disease, osmotic myelinolysis, toxic and metabolic processes, and Creutzfeldt–Jakob disease [3, 6]; which we exclude based on symptoms and above all thanks to the neuroimaging findings.

AOP infarction is an often delayed diagnosis due to various factors. First is a syndrome that is unknown to many emergency physicians due to its rarity, and next, the ultimate diagnosis is typically by magnetic resonance imaging in the hospital course. When we suspect an infarction in the territory of the posterior circulation, it is of vital importance performing a cerebral computerized axial tomography (CT) imaging to exclude hemorrhagic processes and enable adequate treatment with intravenous thrombolysis. Early identification of basilar artery occlusion may allow recanalisation and reperfusion via intravenous and/or intra-arterial thrombolysis. As far as we know, our case is the second case reported along with the one reported by Caruso et al. [7], in which we were able to perform an acute intravenous thrombolysis, often not available because of the delay in the diagnosis. Kostanian and Cramer [8] also reported a case in which they performed acute endovascular thrombolytic therapy for the AOP occlusion that showed clinical and imaging improvement after 24 hours.

Patients have frequently missed the time window for acute management of ischemic stroke, most of the times because of the absence of alterations in the baseline imaging and authors reported a low sensitivity of angiographic study, being brain magnetic resonance the most specific imaging test for diagnosis, often based on clinical suspicion [9, 10].

In conclusion, AOP is an uncommon entity of coma in young patients, being of vital importance for emergency physicians to keep it in the differential diagnosis of coma and altered mental status in young people, in order to exclude other vascular etiologies and metabolic diseases and to establish a treatment for a favorable neurologic outcome.
